# AI-2-dependent gene regulation in *Staphylococcus epidermidis*

**DOI:** 10.1186/1471-2180-8-4

**Published:** 2008-01-08

**Authors:** Min Li, Amer E Villaruz, Viveka Vadyvaloo, Daniel E Sturdevant, Michael Otto

**Affiliations:** 1 Laboratory of Human Bacterial Pathogenesis, Rocky Mountain Laboratories, National Institute of Allergy and Infectious Diseases, The National Institutes of Health, Hamilton, MT 59840, USA; 2 Research Technologies Branch, Genomics Unit, Rocky Mountain Laboratories, National Institute of Allergy and Infectious Diseases, The National Institutes of Health, Hamilton, MT 59840, USA; 3 Laboratory of Zoonotic Pathogens, Rocky Mountain Laboratories, National Institute of Allergy and Infectious Diseases, The National Institutes of Health, Hamilton, MT 59840, USA

## Abstract

**Background:**

Autoinducer 2 (AI-2), a widespread by-product of the LuxS-catalyzed S-ribosylhomocysteine cleavage reaction in the activated methyl cycle, has been suggested to serve as an intra- and interspecies signaling molecule, but in many bacteria AI-2 control of gene expression is not completely understood. Particularly, we have a lack of knowledge about AI-2 signaling in the important human pathogens *Staphylococcus aureus *and *S. epidermidis*.

**Results:**

To determine the role of LuxS and AI-2 in S.* epidermidis*, we analyzed genome-wide changes in gene expression in an *S. epidermidis luxS *mutant and after addition of AI-2 synthesized by over-expressed *S. epidermidis *Pfs and LuxS enzymes. Genes under AI-2 control included mostly genes involved in sugar, nucleotide, amino acid, and nitrogen metabolism, but also virulence-associated genes coding for lipase and bacterial apoptosis proteins. In addition, we demonstrate by liquid chromatography/mass-spectrometry of culture filtrates that the pro-inflammatory phenol-soluble modulin (PSM) peptides, key virulence factors of *S. epidermidis*, are under *luxS*/AI-2 control.

**Conclusion:**

Our results provide a detailed molecular basis for the role of LuxS in *S. epidermidis *virulence and suggest a signaling function for AI-2 in this bacterium.

## Background

Quorum sensing is the cell population density-dependent regulation of gene expression by small signaling molecules, called autoinducers (AI) [1]. Many bacteria have several quorum sensing systems. For example, in the extensively studied *Vibrio harveyi*, there are two classes of quorum-sensing systems, one of which utilizes an acylhomoserine lactone as signal (AI-1), and the other a signal molecule commonly referred to as AI-2 [2]. The biochemical synthesis of AI-2 involves several enzymatic steps starting from *S*-adenosylmethionine (SAM), particularly that catalyzed by LuxS, which produces AI-2 as a side product in addition to the primary role of this enzyme in the activated methyl cycle metabolism [3].

Most quorum-sensing autoinducers are specific for a narrow range of organisms and promote intra-species communication. In contrast, the widely conserved AI-2 has been proposed to allow for communication between species [4]. In fact, more than 55 bacterial species are known to possess a gene homologous to *luxS*, and many produce AI-2 like activities [5]. Since the discovery of AI-2 in *V. harveyi*, many organisms have been shown to regulate genes specifying diverse functions in a *luxS*-dependent manner, such as virulence factors in *Streptococcus pneumoniae *[6], *E. coli *(EHEC) O157:H7 [7], and *Streptococcus pyogenes *[8]; motility in *Campylobacter jejuni *[9], and biofilm formation in *Streptococcus gordonii *[10], *E. coli *K-12 [11], *Bacillus cereus *[12], *Streptococcus mutans *[13], and *Klebsiella pneumonia *[14]. However, the function of AI-2 in most bacteria is not completely understood, owing to the fact that distinguishing between a genuine signal and a mere role as a metabolic side product is difficult [15]. Clear evidence for a signal function can be derived from the discovery of AI-2-specific sensor/regulator systems and transporters. In *V. harveyi*, AI-2 is detected by a two-component system called LuxP/LuxQ [16,17], whose AI-2 dependent activation results in the modulation of gene transcription. However, LuxP homologues are found only in *Vibrio *[18]. In non-*Vibrio *species, the only genes shown to be directly regulated by AI-2 encode an ABC transporter in *Salmonella enterica *serovar Typhimurium named Lsr, which in that species is responsible for AI-2 uptake [19,20].

*Staphylococcus epidermidis *is the most frequent cause of nosocomial sepsis and catheter-related infection [21]. *S. epidermidis *has one well-characterized quorum-sensing system termed *agr *for accessory gene regulator [22,23]. Additionally, like many other bacteria, *S. epidermidis *contains a *luxS *gene and produces AI-2 [24]. In *S. aureus*, inactivation of *luxS *strains does not affect virulence-associated traits, such as the production of hemolysins and extracellular proteases, biofilm formation, and the *agr *system [25]. In contrast, *S. epidermidis luxS *has been shown to influence biofilm formation *in vitro *and enhance virulence in a rat model of biofilm-associated infection [24]. However, whether AI-2 functions as a signaling molecule in staphylococci has remained a matter of debate, mostly because evidence was only derived from the comparison of *luxS *mutants with the corresponding wild-type strains, and sensors or transporters for AI-2 in *Staphylococcus *species are not known. Therefore, to gain further insight into the role of AI-2 in staphylococci, and specifically in *S. epidermidis*, we synthesized AI-2 using *S. epidermidis *Pfs and LuxS enzymes and analyzed AI-2-dependent gene regulation using transcriptional profiling in wild-type, *luxS *mutant, and *luxS *mutant strain with exogenous addition of AI-2. AI-2 regulated genes included genes involved in glycol-, nucleotide, amino acid, and nitrogen metabolism, but also virulence-associated genes coding for the pro-inflammatory PSM peptides, lipase, and the bacterial apoptosis Lrg proteins. Our study suggests that AI-2 has a signaling function in *S. epidermidis *and an important role in the control of metabolism and virulence.

## Results

### Characterization of the *S. epidermidis luxS *mutant

**Figure 1:**
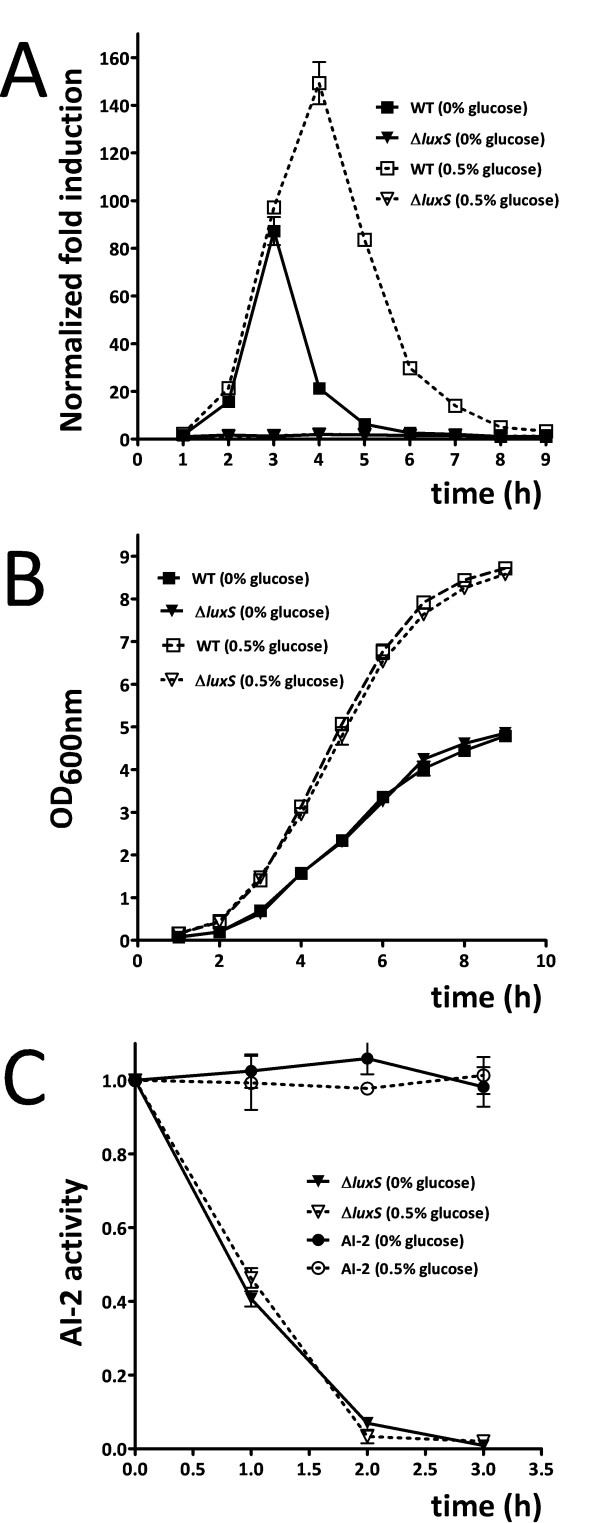
(A) Detection of AI-2 activity during growth of *S. epidermidis *1457 and *S. epidermidis *1457Δ*luxS*. Every hour, cell-free supernatants were examined for the capacity to induce light production in *V. harveyi *BB170. Data were obtained from at least three independent experiments and normalized against light production in TSB or TSB/0.5% glucose. (B) Growth of *S. epidermidis *1457 and *S. epidermidis *1457Δ*luxS *in TSB with different concentration of glucose. Overnight cultures were 1:100 diluted in TSB without glucose or TSB supplemented with 0.5% glucose and the OD_600 nm _during growth of the main cultures was determined. (C) AI-2 activity during incubation with *S. epidermidis *1457Δ*luxS *and in media controls. AI-2 was added to cells of *S. epidermidis *1457Δ*luxS *at exponential growth phase in TSB without glucose and TSB with 0.5% glucose. The same quantity of AI-2 was added to the cell-free culture as control. Afterwards, every hour 1 ml of cell-free supernatant was collected for the detection of AI-2 activity.

First, the isogenic *luxS *mutant strain of strain *S. epidermidis *1457 was characterized for growth and AI-2 production under the conditions used for the subsequent gene expression experiments. As expected, no AI-2 could be detected in the isogenic *luxS *mutant strain, grown in TSB without glucose or TSB with 0.5% glucose (Fig. 1A). In the wild-type strain, AI-2 activity peaked during exponential growth phase and declined during stationary phase. Further, deletion of *luxS *did not affect growth of S. *epidermidis *1457, indicating that its role in metabolism is not essential for bacterial growth under the tested nutrient-rich conditions (Fig. 1B).

### *In vitro *production of AI-2 by purified Pfs and LuxS and *luxS*-independent removal of AI-2 from cultures

For complementation of AI-2 dependent signaling in the *luxS *mutant strain by external addition of AI-2, GST-Pfs and 6His-LuxS were used to synthesize AI-2 from S-adenosylhomocysteine (SAH). Most of His-tag-LuxS was soluble using standard conditions. To obtain soluble GST-Pfs, a low concentration of IPTG was used (0.25 mM), and cultures were grown at low temperature (25°C). Protein functionality was tested via AI-2 production using the *V. harveyi *BB170 bioassay, and induction of the reporter strain was determined as described before (Table 1).

**Table 1:** In vitro AI-2 production from SAH using purified proteins

Substrate	Protein	Normalized fold induction
SAH	None	1
SAH	Pfs	7
SAH	LuxS	12
SAH	Pfs+LuxS	29200
SAH	Pfs+LuxS (filtered)^1^	28700

^1 ^reactions were filtered to remove protein

AI-2 can be removed from culture supernatants in a *luxS*-independent manner, as shown in *Pseudomonas fluorescens *[3], which does not have *luxS*. Similarly, we found that the *S. epidermidis luxS *mutant strain had the capacity to remove AI-2 activity from culture supernatants, while AI-2 in controls was stable over the same period of time (Fig. 1C). It is not clear at this point whether the removal of AI-2 is due to it being metabolized or imported for signaling purposes.

### AI-2 dependent gene regulation in *S. epidermidis*

Genome-wide transcriptional profiling with DNA microarrays was used to determine (i) the *luxS *regulon in *S. epidermidis *1457 and (ii) the extent to which addition of synthetic AI-2 complements alterations in gene expression in the *luxS *mutant (Table 2). We used 2-fold alteration as a cutoff. Most of the AI-2 regulated genes were involved in sugar, nucleotide, amino acid, and nitrogen metabolism, but AI-2 also regulated virulence-associated factors such as lipase, phenol-soluble modulins (PSMs) and the bacterial apoptosis protein LrgB. Furthermore, we observed much stronger changes in gene expression when using TSB without glucose, while the addition of 0.5% glucose in general had similar, but much reduced effects (data not shown). These results may indicate that the metabolic role of *luxS *is more important under nutrient limitation, consistent with results achieved in *S. aureus*, where reduced growth was only found under strong nutrient limitation in sulfur-limited defined media [25].

**Table 2:** Gene regulatory responses in *S. epidermidis *wild-type, Δ*luxS *and Δ*luxS *with exogenous AI-2

**Gene number**	**Gene product**	**Δ*luxS*/WT**	**Δ*luxS*+ AI-2/Δ*luxS***
	**Glycometabolism**		
SE0164	Hexose phosphate transport protein	**0.46**	**4.82**
SE1219	Truncated transposase	**2.41**	**0.44**
SERP1353	Phosphoenolpyruvate carboxykinase	**0.3**	**6.43**
SERP1791	PTS system, lactose-specific IIA component	**0.52**	**2.15**
SERP1792	Tagatose 1,6-diphosphate aldolase	**0.45**	**2.23**
SERP1793	Tagatose-6-phosphate kinase	**0.41**	**2.47**
SERP1794	Galactose-6-phosphate isomerase	**0.4**	**2.45**
SERP1795	Galactose-6-phosphate isomerase	**0.42**	**2.48**
SERP1909	PTS system, IIBC components	**0.37**	**3.88**
SERP2057	Gluconate transporter, permease protein	**0.48**	**3.94**
SERP2058	Gluconokinase	**0.33**	**6.43**
SERP2059	Gluconate operon transcriptional repressor	**0.37**	**6.03**
SERP2100	Ribokinase	**0.49**	**2.71**
SERP2101	Ribose transport protein	**0.42**	**3.14**
SERP2260	PTS system, fructose-specific IIABC components	**0.29**	**8.11**
SERP2261	Mannose-6-phosphate isomerase	**0.34**	**5.9**
SERP2312	Malate:quinone oxidoreductase	**2.64**	**0.33**
			
	**Nucleotide metabolism**		
SERP0067	Xanthine phosphoribosyltransferase	**3.17**	**0.4**
SERP0068	Xanthine permease	**3.35**	**0.39**
SERP0652	Phosphoribosylformylglycinamidine synthase, PurS protein	**3.65**	**0.45**
SERP0653	Phosphoribosylformylglycinamidine synthase I	**3.84**	**0.37**
SERP0654	Phosphoribosylformylglycinamidine synthase II	**3.17**	**0.4**
SERP0655	Amidophosphoribosyltransferase	**3.68**	**0.26**
SERP0656	Phosphoribosylaminoimidazole synthetase	**2.62**	**0.33**
SERP0657	Phosphoribosylglycinamide formyltransferase	**2.52**	**0.35**
SERP0658	Phosphoribosylaminoimidazolecarboxamide formyltransferase/IMP cyclohydrolase	**2.45**	**0.34**
SERP0659	Phosphoribosylamine – glycine ligase	**2.21**	**0.42**
			
	**Amino acid metabolism**		
SERP2128	Delta-1-pyrroline-5-carboxylate dehydrogenase, putative	**0.42**	**3.2**
SERP2327	Acetoin dehydrogenase, E3 component, dihydrolipoamide dehydrogenase	**0.49**	**6.18**
SERP2326	Acetoin dehydrogenase, E1 component, alpha subunit	**0.44**	**5.24**
SERP2325	Acetoin dehydrogenase, E1 component, beta subunit	**0.53**	**4.95**
			
			
	**Nitrogen metabolism**		
SERP1980	Nitrite extrusion protein	**3.16**	**0.28**
SERP1984	Respiratory nitrate reductase, gamma subunit	**2.57**	**0.47**
SERP1985	Respiratory nitrate reductase, delta subunit	**4.81**	**0.39**
SERP1986	Respiratory nitrate reductase, beta subunit	**3.93**	**0.47**
SERP1989	Nitrite reductase	**2.52**	**0.46**
			
	**Other**		
SERP0878	Portal protein, truncation	**3.76**	**0.48**
SERP2027	Antiholin-like protein LrgB	**0.46**	**9.38**
SERP2069	Major facilitator superfamily protein	**0.5**	**2.36**
SERP2222	Transcriptional regulator CadC	**2.09**	**0.48**
			
	**Unclassified**		
SERP0895	Hypothetical protein	**0.36**	**3.53**
SERP1923	Hypothetical protein	**2.27**	**0.43**
SERP2068	Hypothetical protein	**0.45**	**3.99**
SERP2102	Hypothetical protein	**0.37**	**3.84**
SERP2333	Hypothetical protein	**0.53**	**2.55**

**Figure 2:**
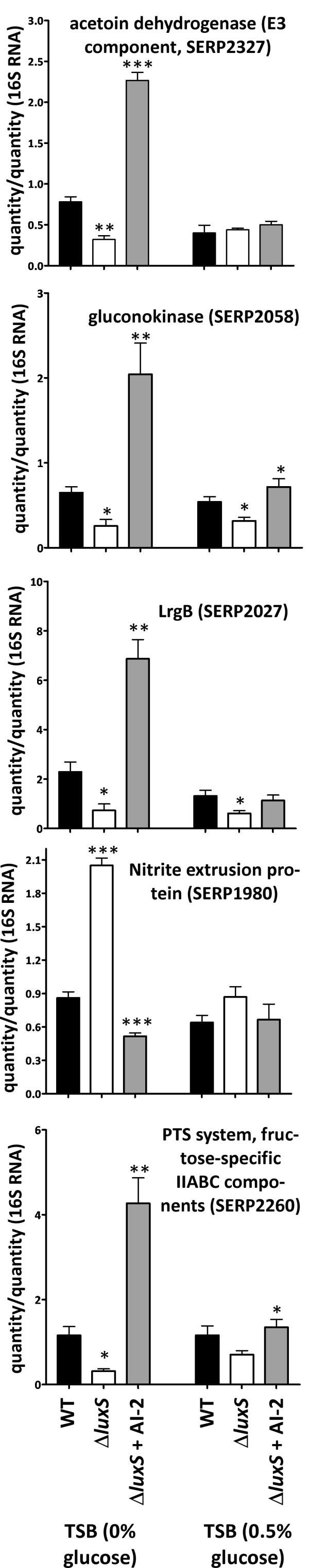
Quantitative RT-PCR of selected *luxS*/AI-2 regulated genes. Growth conditions of cultures in which relative transcription levels were determined were the same as in the microarray experiments. *, p < 0.05, **, p < 0.01, ***, p < 0.001. Comparisons are vs. wild type for Δ*luxS *and vs. Δ*luxS *for Δ*luxS *+ AI-2.

**Figure 3:**
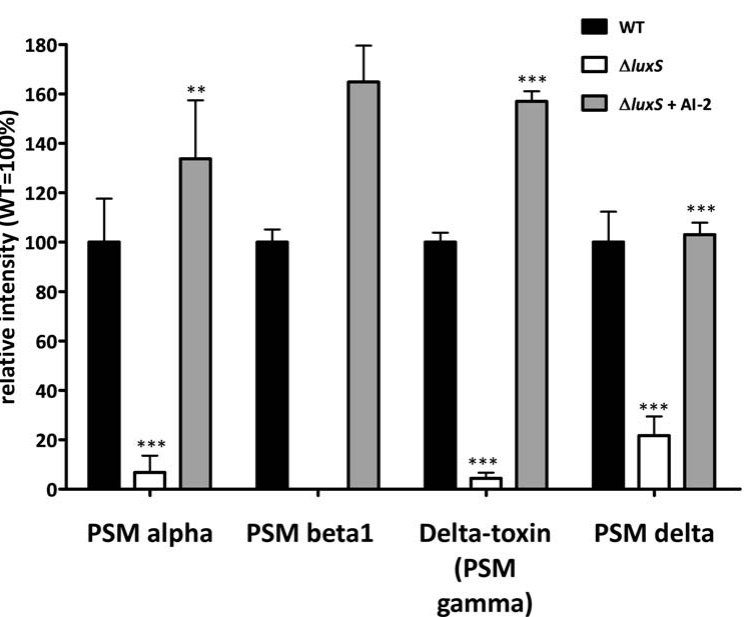
Relative production of major PSMs in wild-type, Δ*luxS*, and Δ*luxS *strain with AI-2 addition. **, p < 0.01, ***, p < 0.001. Data are from the integration of extracted ion chromatograms of the two major peptide peaks produced in electrospray mass chromatograms of culture filtrates using HPLC/MS. Comparisons are vs. wild type for Δ*luxS *and vs. Δ*luxS *for Δ*luxS *+ AI-2. No statistical analysis could be performed for PSM beta1 as there were no detectable levels of PSM beta1 in the Δ*luxS *strain.

To verify our microarray data, we performed real-time RT-PCR on a selected number of the identified AI-2 regulated genes (Fig. 2), which gave results consistent with those achieved using the microarrays. Further, not all PSM genes are included in the DNA microarray owing to their short gene lengths. Therefore, we quantified PSMs in bacterial culture supernatants by HPLC-MS to confirm the impact of *luxS *on the expression of PSM genes (Fig. 3). PSM levels were significantly reduced in the supernatants of the Δ*luxS *strain compared to those obtained from the wild-type strain, and production was restored after addition of synthesized AI-2. These results demonstrate that AI-2 signaling has a very significant impact on PSMs, which represent one of the most important virulence factors of *S. epidermidis *with involvement in both biofilm formation and inflammation [26-28].

## Discussion

In recent years, the skin commensal microorganism *S. epidermidis*, has emerged as a leading cause of hospital-acquired infections [29]. *S. epidermidis *infections are primarily associated with the use of medical devices such as venous catheters. Many regulatory systems control virulence-associated traits in *S. epidermidis *[30]. Specifically, we have recently reported that a *luxS *mutant strain of *S. epidermidis *showed increased biofilm formation in vitro and enhanced virulence in a rat model of biofilm-associated infection [24]. On the contrary, inactivation of *luxS *in various *S. aureus *strains has been reported not to affect virulence-associated traits [25]. In further contrast to *S. aureus*, we show here that AI-2 activity in *S. epidermidis *was not maintained in stationary growth phase, but quickly decreased after obtaining a maximum during exponential growth. Thus, the role of AI-2 in staphylococci remains a matter of debate and there might be species-specific differences.

To gain further insight into *luxS*-dependent gene regulation and AI-2-dependent signaling in *S. epidermidis*, we used genome-wide transcriptional profiling. We synthesized AI-2 with over-expressed *S. epidermidis *enzymes and used the synthesized AI-2 in transcriptional profiling experiments to validate the signal role of AI-2. As main results of our studies, we detected that (i) externally added AI-2 almost completely restored gene expression patterns of the wild-type strain in the *luxS *mutant strain and (ii) *S. epidermidis *regulates virulence-associated factors in addition to metabolism in an AI-2-dependent fashion. Importantly, there was dramatic AI-2-dependent alteration of PSM expression. PSMs have been recently recognized as key pro-inflammatory and immune evasion factors in *S. epidermidis *and *S. aureus *[27,28,31] and very likely have an additional function in biofilm development [26]. Further, we did not observe any influence of *luxS *on *agr*, a quorum-sensing system with a pronounced regulatory effect on PSM expression [28,31] and therefore, *luxS*-dependent regulation of PSMs occurs via a yet undiscovered pathway. Moreover, we observed AI-2-dependent regulation of the antiholin protein LrgB, a possible main player in induced cell death in bacteria and DNA-dependent bacterial biofilm formation [32,33]. Interestingly, we did not find the *ica *genes coding for production of the biofilm exopolysaccharide PIA among the genes regulated by AI-2 under the conditions used (during exponential growth at high activity of AI-2), which contrasts our previous findings that demonstrated *luxS*-dependent control of *ica *during later growth stages [24], when AI-2 activity is low. These findings may suggest that expression of the *ica *genes is impacted by the metabolic function of LuxS rather than AI-2 control, a hypothesis that remains to be validated.

## Conclusion

Our results indicate important species-specific differences in *luxS*-dependent gene regulation between *S. epidermidis *and *S. aureus*. Further, based on the complementation with synthesized AI-2 and the inclusion of virulence genes in the *luxS *regulon, our study suggests that AI-2 has a signaling function in *S. epidermidis*. However, AI-2 signaling in staphylococci needs to be confirmed on a molecular level showing how AI-2 interacts with an external sensor, or alternatively, is imported into the cell for an internal sensor mechanism.

## Methods

### Bacterial strains and growth conditions

The bacteria and plasmids used are listed in Table 3. *E. coli *strains were grown in Luria-Bertani (LB, Oxoid) medium, and *S. epidermidis *strains were grown in tryptic soy broth (TSB, Oxoid). When necessary, antibiotics were added: ampicillin (amp) 100 μg/ml, kanamycin (kan) 25 μg/ml, erythromycin (erm) 2.5 μg/ml. *V. harveyi *BB170 was grown in autoinducer bioassay (AB) medium at 30°C. For microarray experiments, overnight cultures of *S. epidermidis *(wild-type and *luxS *mutant) were 1:100 diluted into 50 ml of TSB and incubated at 37°C with shaking at 180 rpm until grown to mid-logarithmic growth phase. After addition of AI-2 to *S. epidermidis *1457Δ*luxS*, incubation was continued for 1 h.

**Table 3:** Bacterial strains and plasmids used in this study

Strains/plasmids	Relevant genotype and property	Source/reference
*S. epidermidis*		
1457	Wild-type strain	[38]
1457 Δ*luxS*	*luxS *mutant (*luxS*^- ^*erm*^r^)	[24]
*E. coli*		
XL1 blue	*recA1 endA1 gyrA96 thi-1 hsdR17 supE44 relA1 lac *[F' *proAB lacIqZΔM15 *Tn10 (*Tet*^r^)]	Qiagen
SG13009 [pREP4]	K12 derivative, *Nal*^s ^*Str*^s ^*Rif*^s ^*Thi*^- ^*Lac*^- ^*Ara*^- ^*Gal*^+ ^*Mtl*^- ^*F*^- ^*RecA*^+ ^*Uvr*^+ ^*Lon*^+ ^[pREP4 *KanR*]	Qiagen
BL21	F-,*omp*T, *hsd*S(r_B_^-^, m_B_^-^), *gal*	Amersham
*V. harveyi*		
BB170	*luxN*::Tn5(sensor-1^- ^sensor-2^+^), AI-2 reporter strain	ATCC BAA-1117
Plasmids		
pQE-9	*lac*I^q^, 3.4 kb, Ap^r^, T5, C-terminal 6 × His-tag	Qiagen
pQE-*luxS*	pQE-9 containing the *luxS *gene of *S. epidermidis *1457	this study
pGEX-4T-1	*lac*I^q^, 4.9 bp, Ap^r^, GST gene fusion vector	Amersham
pGEX-*pfs*	pGEX-4T-1 containing the *pfs *gene of *S. epidermidis *1457	this study

### Overexpression and purification of LuxS and Pfs

The *pfs *gene was cloned and overexpressed as a glutathione-*S*-transferase (GST) fusion. The *luxS *gene was cloned and overexpressed as histidine residue-tagged (6 × His tag) fusion. Primers for amplification of *pfs *and *luxS *genes from *S. epidermidis *1457 genomic DNA were as follows. For amplification of the *pfs *gene, the primers used were 5'-GCTTTATAAATGAGGTGTGAAAGGATCCATGATAG-3' and 5'-CAATATCTTTTCACCTGAATTCTTATAATGATTCT-3'. For amplification of the *luxS *gene, the primers used were 5'-CAATAAGGAGGATGTCGACATGACTAAAATGAATG-3' and 5'-TTAGTTGTATTGTCTGCAGTTTACCTTCTCCGTAG-3'. PCR products were purified, digested using *Bam*HI and *Eco*RI for *pfs *and *SaI*I and *Pst*I for *luxS*. The *pfs *gene was cloned into the GST gene fusion vector pGEX-4T-1 (Amersham Biosciences), and the recombinant vector pGEX-*pfs *was maintained in *E. coli *strain BL21 (Amersham Biosciences) for overexpression. The *luxS *gene was cloned into the His-tag fusion vector pQE-9 (Qiagen), the recombinant vector pQE-*luxS *was transferred to *E. coli *strain XL1 blue (Qiagen) for propagating plasmids and then transferred to *E. coli *strain SG13009 [pREP4] (Qiagen) for overexpression. Unless otherwise noted, cultures of these two strains were grown at 37°C with aeration to an OD_600 _of 1.0. IPTG was added to a final concentration of 0.5 mM, the cultures were incubated with aeration for an additional 5 h, and cells were harvested. For recombinant Pfs, the fusion protein was purified using the GST-Tag purification Kit (Chemicon) according to the manufacturer's instructions. For recombinant LuxS, the fusion protein was purified on Ni-NTA agarose matrix columns by washing with 10 volumes of 50 mM NaH_2_PO4, 300 mM NaCl, 20 mM imidazole, pH 8.0, followed by elution with 5 volumes of 50 mM NaH_2_PO4, 300 mM NaCl, 100 mM imidazole, pH 8.0. The purified fusion proteins were concentrated in Centriprep-10 concentrators (Amicon) and dialysed against 10 mM sodium phosphate buffer (pH 7.5) using PD-10 Desalting columns (Amersham Biosciences). The sizes of the Pfs GST fusion protein and the LuxS His-tag fusion protein were confirmed by SDS-PAGE.

### *In vitro *production of AI-2

Commercially available S-adenosylhomocysteine (SAH, Sigma) was used as the substrate for AI-2 synthesis [34]. *In vitro *AI-2 synthesis reactions were carried out at 37°C. SAH (1 mM) was incubated with 1 mg/ml purified Pfs in 10 mM sodium phosphate buffer (pH 7.5) for 1 h, and the reactions were filtered through Ultrafree-10 units (Amicon). Subsequently, 1 mg/ml purified LuxS in 10 mM sodium phosphate buffer (pH 7.5) was added, and the reaction mixture was incubated for another hour. After incubation, reactions were filtered through the same filters as described above to remove protein from the reaction product.

### AI-2 bioassays

The AI-2 bioassay that uses the *V. harveyi *reporter strain BB170 was performed as described [35]. Briefly, the *V. harveyi *reporter strain was grown overnight at 30°C with aeration in AB medium, diluted 1:5000 into fresh AB medium, and 90 μl of the diluted cells were added to microtiter wells containing 10 μl of the samples to be tested for AI-2 activity. Sodium phosphate buffer (10 mM, pH 7.5) or medium alone was added as negative control. The microtiter dishes were shaken in a rotary shaker at 180 rpm at 30°C. Every hour, light production was measured using a Microlumatplus LB 96 V luminometer (Berthold). All assays were repeated at least three times.

### *S. epidermidis *microarray experiments

Total RNA was isolated using an RNeasy Mini Kit (Qiagen) as recommended in a standard protocol. In brief, cell pellets were washed with RNase-free water, resuspended in 700 μl of RLT Buffer supplemented with β-mercaptoethanol (10 μl β-mercaptoethanol per 1 ml RLT). The bacterial suspension was transferred to a 2-ml FastPrep lysing tube (Q-BioGene). The cells were lysed in a Bio101 high-speed homogenizer (Savant Instruments), at the following setting: speed, 6.0; time, 20 s. The lysate was incubated on ice for 5 min and centrifuged at 15,000 rpm at 4°C for 15 min. The supernatant was collected and diluted with 500 μl of 100% ethanol. Samples were mixed and transferred to an RNeasy mini column. RNA isolation was performed according to the manufacturer's instructions. Remaining DNA was removed using RNase-free DNase I (Amersham Biosciences). Removal of contaminant DNA was confirmed by PCR. The reaction product was cleaned up with an RNeasy mini column. cDNA was synthesized and labeled according to the manufacturer's suggestions for Affymetrix antisense genome arrays (Affymetrix) as described [36]. A gel shift assay with NeutrAvidin (Pierce Biotechnology) was performed to estimate the labeling efficiency based on the instructions from Affymetrix. Biotinylated *S. epidermidis *cDNA was hybridized to custom Affymetrix GeneChips (RMLChip 3) with 98.9% coverage of genes from *S. epidermidis *RP62A (2467 probe sets of 2494 ORFs) and scanned according to standard GeneChip protocols (Affymetrix). Each experiment was replicated at least 3 times. Affymetrix GeneChip Operating Software GCOS v1.4 was used to perform the preliminary analysis of the custom chips at the probe-set level. Subsequent data analysis was performed as described [36]. To be included in the final gene list, gene expression must have been changed at least 2-fold for one of the treatments. The complete set of microarray data was deposited in NCBIs Gene Expression Omnibus [37] and is accessible through GEO Series accession number GSE9427.

### Quantitative reverse-transcription (RT) polymerase chain reaction (PCR)

Oligonucleotide primers and probes (Tab. 4) were synthesized by Sigma. Probes for quantitative RT-PCR were used to continuously monitor formation of PCR products during PCR. cDNA was synthesized from total RNA using the SuperScript III first-strand synthesis system (Invitrogen) according to the manufacturer's instructions. The resulting cDNA and negative control samples were amplified with TaqMan Universal PCR Master Mix (Applied Biosystems). Reactions were performed in a MicroAmp Optical 96-well reaction plate using a 7700 Sequence Detector (Applied Biosystems). Standard curves were determined for each gene, by use of purified chromosomal template DNA at concentrations of 0.005–50 ng/ml. All RT-PCR experiments were performed in triplicate, with 16S RNA used as a control.

**Table 4:** Oligonucleotide primers and probes used for RT-PCR

Gene product		Oligonucleotide(5'-3')
Acetoin dehydrogenase	Forward	GAGCAAGCTAAAGAGGCTGGTTAT
	Probe	CCTTGAAATGCTGATTGAGTCACTTTCACAT
	Reverse	CCTTTGATTAACGCCTTTGCAT
		
Gluconokinase	Forward	AGTAAAACCAGGTGCAGAAGGATT
	Probe	CGCGCTCTCCAGCTAAATAGGG
	Reverse	CGTCAGCGTTCCACAATGG
		
Antiholin-like protein LrgB	Forward	TGTCGCGGCAGCTAAAGAA
	Probe	ACTGCAATACTTCCCATTGATTCTTCAG
	Reverse	CAACAATAACGCCAACGATGAC
		
Nitrite extrusion protein	Forward	GGTTGGTGGTGTTATAGGTGATAAATTT
	Probe	AAGCGCTTATCATCGACTTCGTG
	Reverse	GCTTAATATAAGCGCACCAATAATCAT
		
PTS system, fructose-specific IIABC components	Forward	TTAATCACAGCAAACAACTCTCACAT
	Probe	CAATGAGTGATTTATCGAACACCATAT
	Reverse	TCTTGTCATTGGTTCCTTGGTAATT

### Detection and quantitation of PSMs in bacterial culture filtrates

High-pressure liquid chromatography – mass spectrometry (HPLC-MS) was used to detect and quantify PSMs in bacterial culture supernatants. One hundred-microliter samples from cultures were injected onto an analytical reversed-phase column (Zorbax C8, 2.1 × 30 mm; Agilent). A gradient from 0.1% trifluoroacetic acid (TFA) in 50% acetonitrile/50% water to 0.1% TFA in 90% acetonitrile/10% water was applied by use of an Agilent 1100 system connected to a VL trap mass spectrometer.

## Authors' contributions

ML performed all research. AEV assisted in protein purification and VV in microarray experiments. DES performed microarray hybridization and analysis. ML and MO conceived the study and analyzed results. MO supervised research and wrote the paper. All authors read and approved the final manuscript.
